# On the Nature of Monozygotic Twin Concordance and Discordance for Autistic Trait Severity: A Quantitative Analysis

**DOI:** 10.1007/s10519-019-09987-2

**Published:** 2019-12-18

**Authors:** Lauren Castelbaum, Chad M. Sylvester, Yi Zhang, Qiongru Yu, John N. Constantino

**Affiliations:** 1 Department of Psychiatry, Washington University School of Medicine, St. Louis, Missouri USA; 2 Department of Pediatrics, Washington University School of Medicine, 660 S. Euclid Avenue, Campus Box 8504, St. Louis, Missouri 63110 USA

**Keywords:** Autism, Development, Heritability, Genetics, Epidemiology, Twin studies, Epigenetics

## Abstract

The characterizing features of autism spectrum disorder (ASD) are continuously distributed in nature; however, prior twin studies have not systematically incorporated this knowledge into estimations of concordance and discordance. We conducted a quantitative analysis of twin–twin similarity for autistic trait severity in three existing data sets involving 366 pairs of uniformly-phenotyped monozygotic (MZ) twins with and without ASD. Probandwise concordance for ASD was 96%; however, MZ trait correlations differed markedly for pairs with ASD trait burden below versus above the threshold for clinical diagnosis, with R^2^s on the order of 0.6 versus 0.1, respectively. Categorical MZ twin discordance for ASD diagnosis is rare and more appropriately operationalized by standardized quantification of twin–twin differences. Here we provide new evidence that although ASD itself is highly heritable, variation-in-severity of symptomatology above the diagnostic threshold is substantially influenced, in contrast, by non-shared environmental factors which may identify novel targets of early ASD amelioration.

**Electronic supplementary material:**

The online version of this article (10.1007/s10519-019-09987-2) contains supplementary material, which is available to authorized users.

## Introduction

In any disease, the concordance rate for identical twins reared together serves as a foundational parameter for estimating heritability. In autism spectrum disorder (ASD), contemporary published reports of probandwise concordance for identical twins have varied from 65 to 90%, (Ronald and Hoekstra 2011; Constantino 2014), in part as a function of the method of ascertainment of identical twins, and in part as a function of variation in the narrowness-of-definition of categorical “case” designation. The generally highly-elevated monozygotic (MZ) twin concordance rate for ASD, in combination with the comparatively low dizygotic twin concordance rate (less than half of the observed MZ twin concordance rate in nearly all previously published twin studies) has established ASD as one of the most heritable of all neuropsychiatric disorders. The discovery, however, that autistic symptomatology exhibits a fully continuous distribution in the general population (Constantino 2011)—and that the distribution reflects highly overlapping causal influence across the range from sub-clinical to clinical affectation (Constantino et al. 2010; Robinson et al. 2011)—raises critical questions about the extent to which calculations of MZ concordance may actually be *underestimated*, and whether previously-reported heritability estimates for sub-clinical severity (Constantino and Todd 2003) extend to measured variation in severity in the clinical range of affectation. This was preliminarily addressed in a population study that implemented diagnostic observations among screen positive MZ twin pairs involving a small number of affected pairs (n < 20) and a substantial number of unaffected pairs (Colvert et al. 2015), among whom severity correlations were observed to be strong in both groups. This observation contrasted with an earlier report of dissimilarity in symptom burden between clinically-affected identical co-twins in a larger sample, when measured according to retrospective accounts of autism severity provided by parents in developmental history interviews using the Autism Diagnostic Interview-Revised (Mazefsky et al. 2007).

The examination and specification of “discordance” in identical twins affords a unique opportunity to examine key differences between affected and unaffected individuals, controlling for familial variation by virtue of genetic uniformity and the equal-environment assumption. Studies of discordant monozygotic twins (pairs in which only one twin has an ASD diagnosis) allows for the identification of possible causes for autism incurred by non-shared environmental influences and/or somatic mutations. The extant literature on the exploration of biological contrasts among identical twins discordant for autism has involved fewer than 150 pairs, *and has never uniformly addressed the issue of what degree of difference between twins, in a quantitative sense, qualifies for discordance*. Mitchell et al. compared 14 pairs of MZ twins, in which 9 pairs of twins were clinically discordant for ASD, to 14 age and gender-matched typically developing singletons. Differences in brain structure, specifically in the prefrontal cortex, corpus callosum, and posterior vermis, were identified as possible neural substrates for the effect of non-shared environmental influence responsible for exacerbating the severity of autistic symptomatology (Mitchell et al. 2009).

Here, we provide what is, to our knowledge, the first quantitative analysis of MZ twin–twin differences in autistic trait severity, using a standardized measure validated with respect to quantitative severity ratings, and uniformly acquired in three available data sets—one of which involves epidemiologically-ascertained subjects, the other two involving clinically-ascertained subjects (total n for the three groups combined = 366 pairs). All subjects were quantitatively assessed using the Social Responsiveness Scale-2 (Constantino and Gruber 2012). We were recently able to demonstrate the scale’s long-term stability coefficients (on the order of r = 0.65–0.90), in a cohort-sequential study (Wagner et al. 2019) tracking quantitative autistic traits from age 3 to 29 years among individuals with and without ASD (*N* = 602). Clinical twin pairs ascertained from one of this study’s two clinical sources were additionally assessed using a standardized diagnostic observation schedule administered by a trained clinician. The goals of this MZ twin study were to determine the extent and nature of discordance in identical twins, whether the strong MZ twin correlations observed for sub-clinical autistic traits extend to autistic symptoms in the clinical range of severity, and whether MZ twin concordance may be underestimated when closely-scoring identical twins fall on either side of an arbitrary dividing line for affectation. Each of these issues carries significant implications for ongoing research on the biology and genetic structure of autism. We hypothesized that MZ twin discordance would be low (less than 10%)—even among pairs suspected-discordant by their parents—when reframing discordance according to a threshold for quantitative “distance” between twins rather than their respective trait levels in relation to an arbitrary cutoff for affectation.

## Methods and materials

### Sample

The current study utilized archival anonymized data from three registries, each containing identical twins who were uniformly phenotyped using the Social Responsiveness Scale (SRS, see below). Epidemiologically-ascertained monozygotic twins, in which zygosity was confirmed using the 27-items Goldsmith Child Zygosity Questionnaire (Goldsmith 1991, Price et al. 2000), (n = 288 pairs, 105 male–male; 183 female–female, ranging in age from 4 to 15 years) were obtained from the Missouri Family Registry (MFR), a birth record registry, in two separate studies at Washington University: the Missouri Twin Study and the Early Reciprocal Social Behavior Study. In the Missouri Twin Study, during the calendar years of 1999 and 2001, 65% of twin births, as traced through public records, were contacted. One parent from each family completed a zygosity interview (response rate: 93.5%) and an initial behavioral assessment of the twins (response rate: 60.7%). Of the responders, a randomized sample was selected to complete the SRS on both of their twins (response rate for completing the SRS for both twins: 84%). The SRS was filled out by 806 parents on twins ages 7 to 15 years: 232 male–male pairs (95 MZ pairs with complete SRS data), 324 female–female pairs (173 MZ pairs with complete SRS data), and 250 opposite-sex pairs. In the Early Reciprocal Social Behavior (ERSB) Study, a longitudinal study investigating the developmental course of RSB at ages 18, 24, 36 and 48 months, toddler twins from the calendar years of 2011 to 2013 were epidemiologically ascertained through the MFR. Of the 330 eligible families, 180 enrolled in the study (Constantino et al. 2017; Marrus et al. 2015). 95 families completed the SRS when the twins reached 48 months old (20 MZ pairs with complete SRS data at age 4 years). Further details of ascertainment and enrollment are provided in Constantino and Todd (2003) (school-aged sample); and Constantino et al. (2017) (preschool sample).

Separate sets of clinically-ascertained pairs, in which one or (usually) both twins had been diagnosed with ASD in the community, were ascertained from the Interactive Autism Network (IAN) registry (n = 23 pairs) and the Autism Genetic Resource Exchange (AGRE, n = 55 pairs). The clinically-ascertained twin pairs ranged in age from 4 to 18 years at the time of assessment; 62 male–male pairs; 16 female–female pairs. IAN is a national internet-based family registry for Autism that is representative of all clinically-affected children and not selective for multiple incidence families (Constantino et al. 2010); For all IAN subjects, zygosity was documented by parent-report questionnaire. AGRE is a national genetic and phenotypic data repository primarily comprised of families with multiple-incidence ASD (Szatmari et al. 2007) in which zygosity was verified by physician records—a concordantly-affected identical twin pair did not satisfy multiplex inclusion unless there was an additional affected sibling in the family, so there was no systematic enrollment bias favoring concordant over discordant identical twins. Sample characteristics are detailed out in the Supplemental Materials Table S1.

### Measures

#### The Social Responsiveness Scale

The SRS (Constantino and Gruber 2012) is a standardized 65-item measure of quantitative autistic traits, which capitalizes on observations of children in naturalistic social contexts, by parent report. Its internal consistency is very high (alpha = ~ .95), and it distinguishes ASD-affected individuals from controls with a Cohen’s d effect size of ~ 2.7 (Hus et al. 2013). Parent-report data in epidemiologic twin samples has exhibited minimal effects of rater bias or rater contrast, and comparisons of maternal ratings of identical versus non-identical twins in the general population have demonstrated robust heritability of the measure (Constantino and Todd 2005). The SRS and SRS-2 have identical item content; the latter provides updated norms which were used to generate all T-scores reported and analyzed in this study, and characterizes variation in the two DSM-5 domains of ASD: social communication and interaction (SCI) and restricted interests and repetitive behaviors (RRB). Prior studies of the SRS in clinical and epidemiological populations have established that these two subdomains encompass a unitary factor structure (Constantino et al. 2004; Frazier et al. 2012). With a test-retest stability of .91 (Wagner et al. 2019), trait stability measured by the SRS within an individual compares favorably to that of IQ (estimates around .63, see Plomin and Deary 2015) and antisocial behavior (estimates around .50, see Murray and Farrington 2010), for subjects with and without ASD.

For the current sample, density plots of the SRS T-scores for clinical and general population monozygotic twin pairs (depicting one twin for each identical pair only) were constructed to confirm their representativeness of the range for clinical and non-clinical scores that have been extensively reported in prior publications and the SRS-2 Manual (Constantino and Gruber 2012; Frazier et al. 2014a)—these are provided in Supplemental Fig. S1; Standardized (T) scores of epidemiologically-ascertained twins ranged from 37 to 80T with a mean of 49T and a standard deviation of 7.6T. Clinically-ascertained twins ranged from 35 to 128T with a mean of 78T and a standard deviation of 18.5T (therefore, *greater* variance within which to test twin–twin similarity). Both exhibited continuous distributions, with the epidemiologically-ascertained twins representing the non-pathological end of the population distribution and the clinically-ascertained twins strongly shifted toward the pathological end as a group.

#### **Autism Diagnostic Observation Schedule** (Lord et al. 2000)

As an independent measurement of autistic trait severity in the clinically-affected twin pairs ascertained in the AGRE registry, we analyzed ADOS social affect scores—the principal parameter of social deficit severity generated by the scale, from assessments performed on the twins in that sample by clinicians trained and research-certified to conduct and score the observations. A previously published longitudinal study of the ADOS involved 1026 assessments conducted serially among 345 clinical subjects over the period from age 2 to 15 years, and observed that over 80% fell in a latent class of persistently elevated scores for social affect (Gotham et al. 2012).

Higher scores on both the SRS and ADOS indicate greater degrees of quantitative autistic trait burden.

### Data analysis

We first examined the distribution of absolute twin/co-twin differences and its association with age across the respective sub-samples. Given that the SRS-2 has been extensively normed for the general population, we elected to use T-scores whenever possible in order to reflect the magnitude of twin–twin contrasts in a manner that has straightforward and widely-recognizable clinical interpretation; especially for observed contrasts for many of the twins that represented very significant variations in symptomatology and level of function. We constructed scatter plots and computed identical twin–twin correlations. Next, for the clinically-ascertained pairs, twin/co-twin contrasts were plotted in relation to a standard cutoff for clinical-level of affectation for ASD. For the AGRE sample, ADOS scores and diagnostic classifications were used to determine the extent to which quantitative trait differences from the SRS ratings corresponded with expert clinician observation and related to categorical discordance. A simulation sample of SRS scores from archival data of singletons, encompassing the full range of the SRS trait distribution, was constructed to generate pairings whose correlations were equally constrained to the observed identical twin correlation for our entire sample. This was used to generate expectations for correlations at the pathological end of the distribution, against which to compare the observed correlations for clinically-affected MZ twins. The simulated dataset was constructed using R (version 3.5.2); details are provided in Supplementary Materials.

## **Results**

**Fig. 1:**
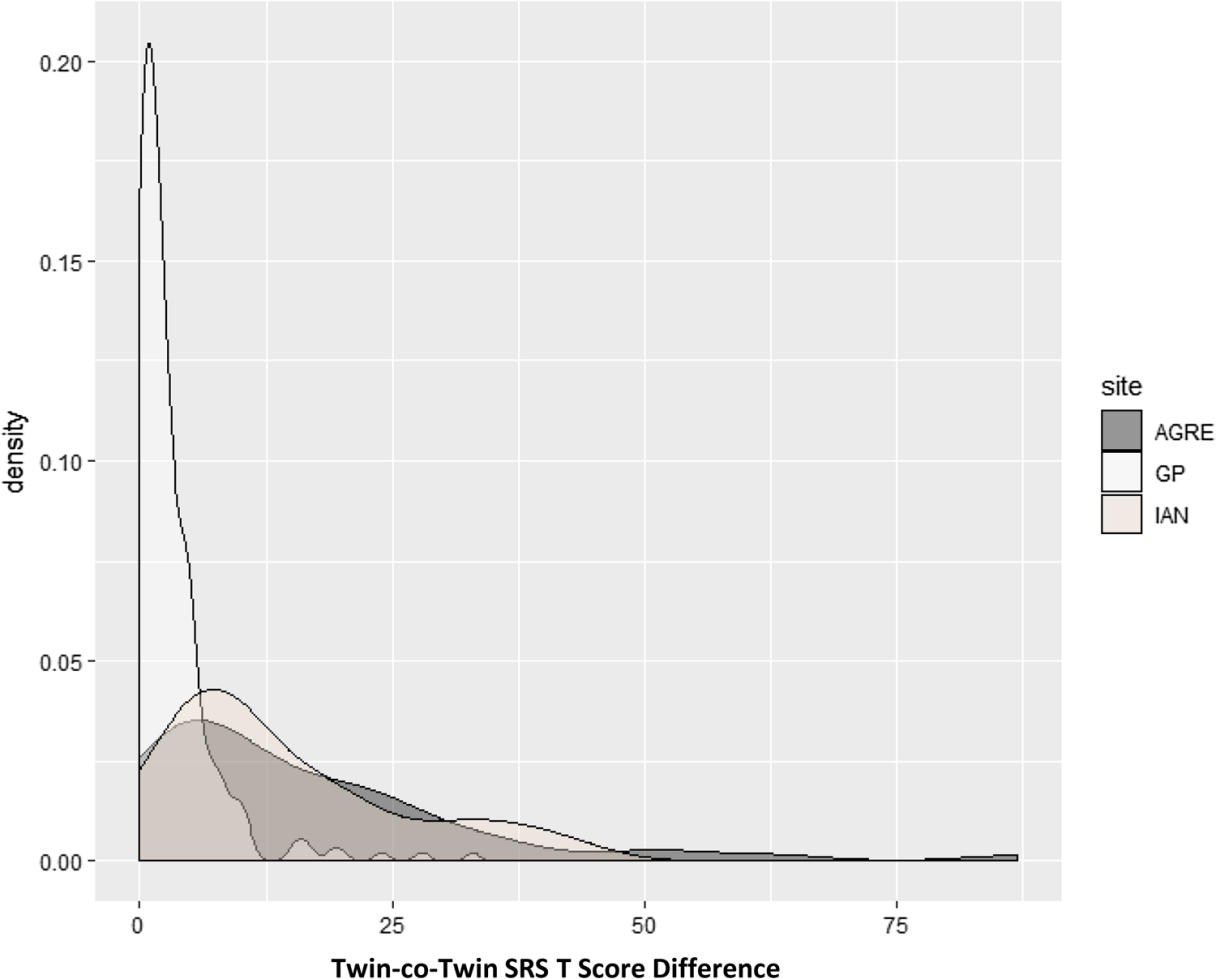
Distributions of MZ twin-co-twin differences for autistic trait severity in superimposed density plots for the three respective samples in the study

Among the monozygotic twin pairs affected by ASD in this sample, the categorical probandwise concordance was 0.91 for the AGRE sample (in which diagnosis for each child was confirmed or excluded by comprehensive research assessment, including expert clinical observation on the ADOS), 0.61 for the IAN sample (in which diagnosis for each twin was reported by parents on the basis of community diagnosis), and 0.82 for the combined clinical monozygotic twin sample. The distributions of MZ twin-co-twin differences for autistic trait severity in the three samples are depicted in Fig. 1. In each subgroup, twin–twin differences were continuously distributed, but we observed marked differences between the epidemiologic and clinical samples in the range and shape of the distribution. The epidemiologically-ascertained, unaffected pairs manifest an extremely narrow distribution of differences by parent report and the pairs affected by ASD manifest a wide range of differences from near zero to pronounced contrasts which occurred far more frequently than observed in the epidemiologic sample. We note that there was substantial overlap in the respective distributions of individual SRS scores for epidemiologically-ascertained and clinically-ascertained twins (as shown in Supplemental Fig. S1).

The mean SRS *difference* for epidemiologically-ascertained pairs was 7.7 (SD 10.1) and did not differ as a function of sex or age of the twins (Pearson’s r = .007; Spearman’s rho = .013), but did differ as a function of mean SRS score of the twins (see below). The mean difference for the clinically-ascertained pairs (all combined) was 33.0 (SD 31.6); in this group, the association with age was Pearson’s r = .089 and Spearman’s rho = .004, indicating that the non-shared environmental influences driving the identical twin–twin differences were not increasing with age over the period from 4 to 18 years. The equivalence in the range of differences identified in the IAN and AGRE samples makes it likely that the MZ twin concordance rate reported by parents for community diagnosis within IAN is an underestimate, since AGRE pairs with comparable levels of difference presumed discordant by their parents were more commonly confirmed to be concordant for ASD when research diagnostic observations were conducted on each twin. For the five AGRE pairs who were confirmed *discordant* on the basis of research diagnostic observation, plots of SRS and ADOS scores are presented in Supplemental Fig. S2, depicting marked differences on both instruments, with the mean for unaffected twins falling below the mean for the general population sample.

**Fig. 2:**
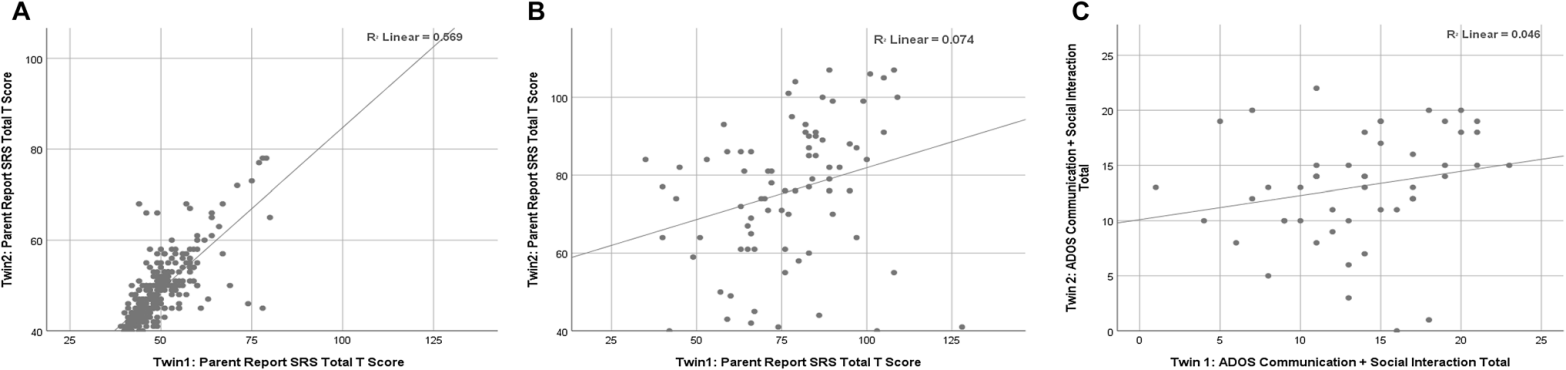
Scatter plots of MZ twin-co-twin data: **a** General population, Social Responsiveness Scale (SRS) scores; **b** Clinically-ascertained MZ twins, SRS; **c** Clinically-ascertained MZ twins, Autism Diagnostic Observation Scale (ADOS)

Scatter plots of the quantitative autistic trait T-scores of the twin pairs are presented in Fig. 2, first for the epidemiologic sample (SRS), next for all clinically-ascertained pairs (SRS), and finally for ADOS scores of the AGRE MZ twins, with coefficients of correlation computed for each plot—this allowed quantification of the variance attributable to familiality (the sum of genetic and common environmental influence) in each respective sample. Remarkably, whereas the identical twin correlation for epidemiologically-ascertained twins was strong, with familial influences accounting for 57% of the variance in quantitative autistic trait scores, the correlations for clinically-ascertained twins were weak, accounting for 7% (SRS) and 5% (ADOS), respectively, of the variance in quantitative trait burden. We know that for the epidemiologically-ascertained sample, there was no difference in the degree of MZ twin-twin correlation between sexes. (For male pairs, Pearson’s r = .746, Spearman’s rho = .800; for female pairs, Pearson’s r = .758, Spearman’s rho = .761; combined, Pearson’s r = .754, Spearman’s rho = .782). The relative non-association of the scores of co-twins among ASD-affected pairs was particularly driven by one of the two principal (DSM5) sub scales of the SRS-2: social communication and interaction (SCI—the other being restricted interests and repetitive behaviors RRB, which is highly inter-correlated with SCI throughout the population distribution, and difficult to reliably ascertain in time-limited clinical observations using the ADOS). Whereas the identical twin correlation for both sub scales among epidemiologically-ascertained twins was robust, with familial influences accounting for 57% (SCI) and 48% (RRB) of the variance respectively, familial influences for clinically-ascertained MZ twins accounted for only 6% of the variance for SCI, with a somewhat higher level observed for RRB (20%). Scatterplots of the Social Communication Index and Restricted Interests & Repetitive Behaviors Index items for both the epidemiologic and the clinical populations are presented in Supplemental Fig. S3. There was no appreciable association between the mean severity of autistic trait burden in a clinically-ascertained pair and the degree of difference between twins (Spearman’s rho = − 0.20 *ns*). In contrast, among the epidemiologically-ascertained twins, those pairs whose average score was nearest to the clinical threshold for affectation tended to show greater twin–twin differences (Spearman’s rho = 0.33, p < .001), in keeping with the notion that the differences become pronounced near the clinical threshold, and uniformly when the clinical threshold has been exceeded (see Supplemental Fig. S4). The identical twin–twin correlation for the entire sample (all groups) was 0.77.

**Fig. 3:**
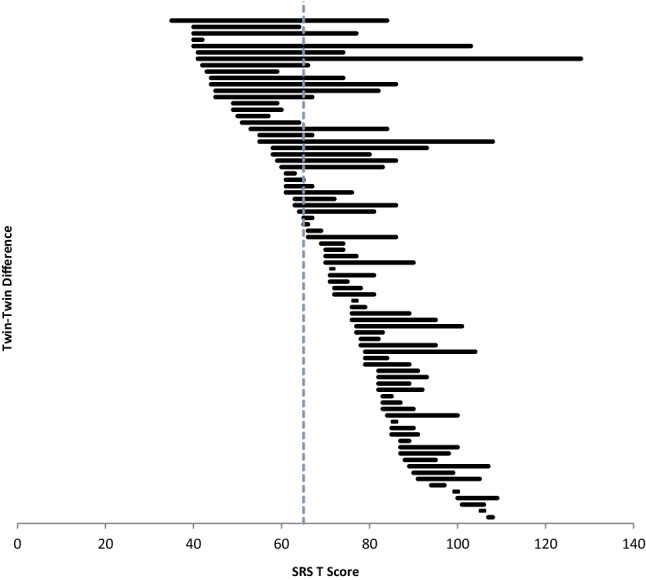
Distribution of clinical MZ twin–twin SRS scores in relation to an established cutoff for clinical-level affectation

To further explore the question of how the *degree* of quantitative MZ twin–twin difference relates to the notion of “discordance” for categorical case designation, we plotted the SRS scores of each pair of twins against an established cutoff for clinical affectation, arranging the display in ascending order of the T-score of the lowest scoring twin (Fig. 3). The plot recapitulated the quantitative nature of twin discordance across the range of severity from sub- threshold to severe clinical affectation; 13 of the lower-scoring twins in clinically-ascertained pairs scored in the sub- threshold range between 50T and the 65T cutoff, and with their co-twins above the cutoff. This subgroup of pairs “straddling the line” of clinical affectation comprised 16% of the MZ twin sample, exhibited only moderate twin–twin differences (typically on the order of one standard deviation or less), and would technically have been considered discordant even though the unaffected twin manifested higher than average quantitative autistic trait burden. Moreover, of the nine pairs from the IAN registry who were reported by their parents as discordant for community diagnosis of ASD, the lower scoring twin in three of the pairs had an SRS score exceeding the clinical cutoff, and four other pairs failed to have a difference score meeting one full standard deviation (18.1) as calculated in the clinical population. Implementing a correction based on these quantitative standards, IAN’s probandwise concordance rate was .91, equivalent to that of AGRE, in which the diagnoses were confirmed by research diagnostic observation. Details of an illustrative clinical case, in which preschool identical twins presumed to be discordant by their parents were comprehensively evaluated in an extension of the research program involving the epidemiologically-ascertained sample, are provided in the Supplementary Materials section Table S2.

Finally, we conducted a simulation to determine the degree of erosion in twin–twin correlation that might be *expected* when sampled from a truncated distribution at the pathological end, and to compare the expected versus the observed results. A simulated dataset of 10,000 twin pairs was generated by sampling with replacement from the SRS scores of 6000 individuals from 2000 large sibship families epidemiologically-ascertained from the State of Missouri (Ramtekkar et al. 2010). Twin pairs were constrained to have a twin–twin correlation of 0.77 in keeping with the magnitude of the correlation observed in the current sample for all subjects combined. When imposing a stringent truncation boundary at a cutoff of 65T (i.e. removing all pairs in which either twin fell below the threshold displayed in Fig. 3), the expected correlation was 0.15, whereas the observed correlation in the actual MZ twin data set for this conservatively-defined sub group was 0.01.

## Discussion

In autism spectrum disorder (ASD), prior reports of concordance in identical twins have been predicated on the presence or absence of a categorical diagnosis in relation to a relatively fixed, or arbitrary, diagnostic threshold. Since it is now known that the characterizing features of the condition are continuously distributed in nature (Constantino 2011, Wagner et al. 2019), and that its marked heritability is substantially mediated by additive/polygenic risk (Sandin et al. 2017; Weiner et al. 2017), we conducted a quantitative analysis of autistic trait variation in a large sample of identical twins, representing the entire range of the variation in autistic trait burden observed in nature. The data overwhelmingly supported extremely high heritability for the condition itself, by virtue of confirming a very high probandwise concordance rate (on the order of 0.90) across both samples of clinically-ascertained twins. Recent analyses of twin and family data from very large epidemiologically ascertained samples have estimated the heritability of autism at 0.85 (Sandin et al. 2017).

When considering quantitative measurements of autistic severity, we observed a monozygotic twin correlation of 0.76 among epidemiologically-ascertained twins and a very tight distribution of twin–twin differences, averaging 8 raw score points which translates to less than one half of a standard deviation of the population mean, in keeping with prior reports of the heritability of sub-clinical autistic traits in the general population (Constantino 2011). Marked differences in clinical severity were observed; however, between clinically-affected identical twins; these differences exhibited a broad continuous distribution, essentially equivalent in the two independent clinically-ascertained samples, with mean twin–twin differences four times higher than those observed in the epidemiologically-ascertained pairs. Although SRS score differences at the higher versus lower range of the population distribution may not be directly comparable, what we observed among the twins was clinically significant by any standard—as a point of reference, treatment studies of clinically-affected patients consider a change in 10 points to be evidence of efficacy (see Anagnostou et al. 2015). Among identical twin pairs in which one or (usually) both carried a diagnosis of ASD, the twin-co-twin difference averaged 33 points on the SRS, and  the trait correlations were much lower than in the epidemiologic sample, 0.27 when measured by the SRS and 0.21 when measured by the ADOS, with twin–twin contrasts more pronounced for social communication and interaction than for restricted interests and repetitive behaviors.

In order to determine whether these erosions in MZ twin–twin correlations in the clinical range of severity might be accounted for by mathematical effects of sampling from the pathological range of a distribution, we conducted a simulation in which correlations were computed exclusively from pairs lying above a conservative line of truncation of the sample (65T). In this restricted range, the observed correlations fell far below what would be expected mathematically. In reality, the distributions of quantitative trait scores for the clinical and non-clinical samples exhibited substantial overlap extending well into the non-pathological range of the distribution; in this sense, there was no clear truncation of the distribution on which to base an absolute threshold to simulate. When considering, however, that ascertainment of clinical cases is based on a general threshold of impairment, another reasonable approach to establishing mathematical expectations would be to consider a range of less stringent latent thresholds. When considering cutoffs across the range from the upper 70% to the upper 30% of scores for two normally-distributed traits with correlation 0.75 (as observed in this sample), published expected bivariate correlations range from 0.50 to 0.62 respectively (Johnson and Kotz 1972). Again, our observed correlation coefficients fell well below these values. Also, there was no effect of erosion of twin–twin correlation when considering exclusively the non-clinical sample (which, in essence, reflected more truncation in relation to the pathological range than the clinical sample did in relation to the non-pathological range). We note also that a previous study of supra normal intelligence, restricted to MZ twins above the 85th percentile for IQ exhibited no significant erosion of the identical twin correlation in comparison to that observed for IQ in the general population (Haworth et al. 2009). Body mass index reflects a counterexample in which differential heritability at the tail of the distribution has been documented and is believed to reflect distinct environmental influences that come on line in that range (Tsang et al. 2018).

Although these findings await replication in a sample comprising an even larger number of identical twins in the clinical range of affectation, an overarching interpretation of these results is that the factors responsible for variation in severity within the clinical range *diverge from those that are responsible for the heritability of the condition itself*. The inference is that when genetic liability for ASD exceeds a clinical threshold, it engenders a marked increase in vulnerability to the effects of non-shared environmental influences, which were observed to exert minimal effects on individuals whose ASD trait burden fell below the threshold for diagnosis.

Recently, Hegarty et al. (2019) observed greater environmental influences on some aspects of brain morphometry (cortical thickness and cerebellar white matter volume) in twins with ASD than in normal twins; however, most of the observed effects were attributable to shared rather than non-shared environment. The latter dominated our observations and could encompass epigenetic changes, random developmental (in utero) or environmental (extrauterine) perturbations, or the effects of somatic mutations, to which affected individuals might be more vulnerable than unaffected individuals. We note that in the latter case, a relatively high and influential somatic mutation rate would need to be invoked to explain the continuous nature of the distribution of (accentuated) twin–twin differences observed in our clinical sample. Overall, these findings constitute an identical twin demonstration of a remarkable observation originally reported by Spiker et al. (2002) that even in the context of familial recurrence of autism, the specific profile of symptoms among affected individuals do not breed true within families. Very recently, Gazestani et al. (2019) observed transcriptomic signatures of leukocytes that differentiated children with autism from typical controls and related to variation in expression of genes implicated in the cause of autism—such epigenetic variation, if replicated, could harbor clues to the mechanisms underlying stochastic and/or non-shared environmental influences on variation-in severity implicated by this study.

The general notion that disease states incur disruption of expected relationships between biological systems is consistent with theories of typical development that invokes the concept of “canalization,” in which behavioral trajectories *stabilize* as development proceeds and are progressively less prone to the effects of random perturbations (Shultz et al. 2018). In contrast, when variation at the pathologic extreme disrupts this process, a direct consequence may be vulnerability to such perturbations and an increase in variance as a signature of atypicality—this has been observed in the pleiotropic effects of other deleterious influences on developmental phenotypes, including patterns of variation in gene expression across individuals affected by discrete monogenic syndromes engendering autism (Nishimura et al. 2007). In this study, the notable absence of age effects on the substantial non-shared environmental factors influencing severity ratings of identical twins, coupled with knowledge from prior studies of the very high longitudinal stability of severity measurements *within an affected individual* from early childhood through adulthood (Wagner et al. 2019) suggests that the observed non-shared environmental influences on clinical severity may be exerted early in development (prior to age 4 years, for which we document a case example in Supplemental Materials section 7), and that these would constitute early targets (in utero or in infancy) for preventive interventions to ameliorate severity in the setting of clinical-level affectation. This interpretation must be considered cautiously since our data were cross-sectional in nature and would need to be followed by demonstration of early effects in a prospective longitudinal context. It is possible that the identification of a severity threshold at which vulnerability to non-shared environmental influence becomes amplified might serve as a meaningful cutoff for categorical designation of clinical “impairment” in continuously-distributed disease traits, i.e. not only for autism but for other neuropsychiatric syndromes. Future studies should explore whether this general phenomenon may contribute to the formidable—but as yet largely unexplained—influence of non-shared environmental factors observed in genetic epidemiologic studies of many psychiatric disorders.

Our sample size of identical twins in which one or both was affected by clinical ASD was necessarily limited in size and clinically-ascertained. To our knowledge, there does not yet exist an epidemiologic sample collection large enough from which greater numbers of quantitatively-characterized identical twins with clinical-level ASD affectation can be derived. Our trait correlations for clinically affected MZ twins were substantially lower than those obtained from severity measurements in comparably-affected identical twins ascertained in the TEDS UK study via population screening (Colvert et al. 2015); however, in the latter, the number of clinically-affected cases was low (involving less than 20 pairs) and the severity correlation was derived from a mix of diagnosed and undiagnosed twin pairs; it would, therefore, be expected to generate higher MZ co-twin correlations by virtue of the inclusion of subjects below the clinical severity threshold for diagnosis. Although it is possible that clinical ascertainment in this study could have resulted in selection for more discordance than is representative of the population of MZ twins, the categorical probandwise concordance for the sample was over 0.90, the ratio of MZ to DZ twin enrollees in the respective registries was not significantly lower than would be expected in the general population (AGRE–MZ:DZ = 256:458 = .55; IAN– MZ:DZ = 196:737 = .27), and clinical ascertainment afforded a very broad representation of symptom burden including MZ twin pairs who were very severely affected. Furthermore, equivalent continuous distributions of twin–twin differences, observed in our two independent clinical samples, mitigated concerns about the effects of specific ascertainment strategies, and suggested that an increase in sample size would be unlikely to yield fundamentally different results.

Another potential limitation is that only a subset of the identical twins in this study had zygosity confirmed by molecular genetic analysis; however, the high probandwise concordance (again, on the order of 0.90) (Sandin et al. 2017), is highly consistent with estimates for monozygotic twins derived from dedicated twin studies and inconsistent with any significant inclusion of misclassified dizygotic pairs (among whom the epidemiologic probandwise concordance for ASD is on the order of 0.20) (Frazier et al. 2014b; Rosenberg et al. 2009). Some of the observed non-shared environmental influence on clinical twin severity ratings could have occurred on the basis of measurement error, but this was not observed in the epidemiologic sample and a recent analysis of test-retest reliability for maternal ratings of clinically-affected subjects using the SRS-2 (Wagner et al. 2019) demonstrated one-year stability coefficients on the order of 0.90. For the most extreme differences observed in the present cohort, ratings provided by mothers were corroborated by ratings derived from expert clinician observations, making it unlikely that rater contrast effects disproportionately influenced the measurements of clinically-affected MZ twins.

These and other convergent findings have a number of key implications for ongoing research in autism and related disorders. Studies attempting to relate neural or behavioral signatures to genetic susceptibility for ASD may have substantially higher statistical power when conducted among individuals with total symptom burden *below* rather than *above* the clinical threshold for diagnosis. Symptom severity ratings derived from behavioral observations of individuals clinically-affected by ASD, though stable over the course of development and highly reliable in a psychometric sense, may bear little to no relation to the causal (genetic) underpinnings of the condition itself, should not be construed as heritable unless proven to be so, and likely reflect, at least in part, the consequences of random events (to include neonatal medical events) that have disrupted otherwise predictable developmental trajectories, possibly early in life (Willfors et al. 2017). Two prior studies involving alternate measures of autism-related symptomatology in concordant ASD-affected siblings yielded findings that are highly congruent with our observations. Mazefsky et al. (2007) acquired data from the Autism Diagnostic Interview-Revised (ADI-R, a parent-report developmental history measure) that had been obtained on 1294 ASD-affected children in multiplex autism families enrolled in AGRE, and conducted structural equation modeling of the data from twin and sibling pairs. They observed that 72% of the variance in social interaction scores on the ADI-R was attributable to unique environmental influences, leading the authors to suggest at the time that the “etiology of autistic symptom domains” might differ in clinically-affected versus unaffected individuals. Similarly, Goin-Kochel et al. (2008) examined phenotypic congruence in concordant affected AGRE siblings on measures of intelligence (n = 226 families) and adaptive functioning (n = 348 families). Sibling correlations for parent-reported adaptive functioning in the social and communication domains were on the order of 0.20, whereas those for motor function were on the order 0.60, and those for measured cognitive function were intermediate, on the order of 0.40.

If our findings on the relative non-heritability of severity ratings among clinically-affected cases are representative of most or all autistic syndromes, any observed relationships between biological markers and severity ratings would not be expected to relate to the cause of the condition, rather to modifiers or epiphenomena, and, therefore, should be interpreted cautiously. Moreover, these data underscore alternate prospects for biomarker discovery in which neural, genetic, and physiologic signatures are linked to endophenotypic contributors to autistic syndromes (see Pohl et al. 2019; Constantino 2019) within the normal range of the population distribution.

Finally, these data qualify understanding of the foundational statistic of monozygotic twin concordance in autism. Our findings confirm probandwise categorical concordance statistics consistent with marked heritability for ASD (Sandin et al. 2017); although dizygotic twins were not included in this study, our analysis of MZ pairs served to parameterize upper limits on the estimation of heritability for clinical severity measurements, and to confirm the estimates of categorical concordance from classic twin studies that have been foundational in estimating the heritability of autism in the general population (Frazier et al. 2014b). Identical pairs in which one or both exceed the clinical threshold for affectation exhibit a continuous range of contrasts, the most pronounced of which qualify for categorical discordance, but this occurs rarely. More often, even when there exist substantial differences between twins, both typically exhibit levels of symptom burden near or above the threshold for a diagnosis. The broad range of quantitative twin–twin differences documented in this study account for the wide variations in previously reported statistics for MZ twin correlation, particularly from small samples in which concordance was defined in relation to an arbitrary threshold. Based on the current findings, we favor a more standardized parameterization of identical twin *discordance* that is quantitatively defined, on the basis of (1) at least a 1.5 standard deviation difference between twins, *and* (2) the members of the pair falling on opposite sides of an established clinical severity threshold.

## Electronic supplementary material

Below is the link to the electronic supplementary material.

Electronic supplementary material 1 (DOCX 264 kb)
