# Supplementary material for: On the Nature of Monozygotic Twin Concordance and Discordance for Autistic Trait Severity: A Quantitative Analysis
Source: Behav Genet. 2019 Dec 18;50(4):263–72. doi: 10.1007/s10519-019-09987-2 (PMC7355281; doi:10.1007/s10519-019-09987-2)
Supplement: Supplementary file 1 — Electronic supplementary material 1 (DOCX 264 kb) [file 10519_2019_9987_MOESM1_ESM.docx]

**Supplementary Materials**

Table of Contents

Supplemental Table S1- Sample Characteristics1

Supplemental Figure S1- Density plot of SRS T score 2

Detail Description of the simulation sample2

Supplemental Figure S2 - SRS and ADOS scores of 5 AGRE twin pairs 3

Supplemental Figure S3 - Scatter plots of MZ twin-co-twin data 4

Supplemental Figure S4 - Scatter plot of the SRS score 4

Supplemental Table S2 - Illustrative case 4-6

Reference 6

1. Supplemental Table S1.

**Sample Characteristics**

|  | **Total Epidemiologically- Ascertained MZ Twins**  **(N=288)** | |  | | | | |  |  |  |  |
| --- | --- | --- | --- | --- | --- | --- | --- | --- | --- | --- | --- |
|  |  |  | **Total Clinically-Ascertained MZ Twins**  **(N=78)** | | | | | | | |  |
|  | | | | | | | | | | | |
|  | **%** | **N** | **%** | | | | **N** | | | |  |
| **Sex** |  |  |  | |  | |  | | |  |  |
| Male | 36.5 | 105 | 79.5* | | | | 62* | | | |  |
| Female | 63.5 | 183 | 20.5* | | | | 16* | | | |  |
|  | | | | | | | | | | | |
| **Age Range** | 4-15 years old | | 4-18 years old* | | | | | | | | |
|  | | | | | | | | | | | |
|  | Total Epidemiologically-Ascertained MZ twins | | IAN Participants  Community Diagnosis | | | AGRE Participants  Research Diagnosis | | | | | |
| **Total** | 288 | | 23 | | | 55 | | | | | |
|  | | | | | | | | | | | |
| **Zygosity Confirmation** | Zygosity Interview | | Parent Report | | | Verified by Physician Records | | | | | |
|  | | | | | | | | | | | |
| **ASD Diagnosis** |  |  | **%** | **N** | | | \| **%** \| **N** \| \| --- \| --- \| | | | |  |
| Discordant |  |  | 39.1 | 9 | | | 9.1 | | 5 | |  |
| Concordant |  |  | 60.9 | 14 | | | 90.9 | | 50 | |  |
| **Abbreviations:** AGRE = Autism Genetic Resource Exchange; IAN = Interactive Autism Network; MZ = monozygotic; * calculated for the Total Clinically-Ascertained MZ Twin sample | | | | | | | | | | | |

1. Supplemental Figure S1. Density plot of SRS T score (one twin selected at random per pair) for epidemiologically-ascertained and clinically-ascertained monozygotic twin pairs, depicting the severity range and continuity of the distributions in the respective sub samples.


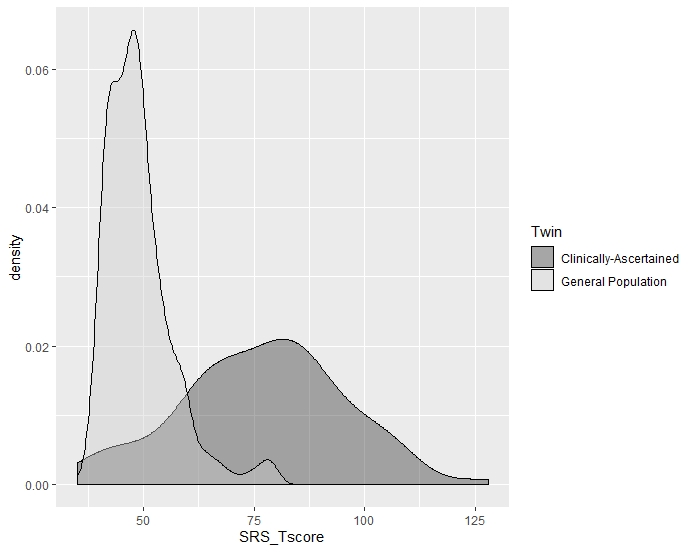


SRS T-scores

1. Methods for derivation of a simulation sample in which to conduct a conservative test of expected correlation when sampling from the pathological end of an SRS distribution

A sample of 10,000 individual SRS scores was generated by sampling with replacement from the SRS scores of 6000 individuals in 2000 families enrolled in an epidemiologically-ascertained large sib-ship sample from the State of Missouri (Ramtekkar UP, et al 2010). Separately, a second sample of 10,000 SRS scores was derived in the same manner to use as starting values for the twins for each of the initial 10,000 simulated participants. We next a) derived the shared and residual components of the variance between the initial sample and the second ‘twin’ sample; b) computed the twin scores from the initial 10,000 SRS scores and the residual components using Cholesky decomposition to constrain the twin-twin correlation in SRS scores to be 0.75 across the entire distribution, and finally c) standardized the twin scores to have the same standard deviation and mean as the initial 10,000 individual SRS scores. In this simulated sample of SRS scores in which the bivariate correlation was fixed at 0.75, the correlation coefficient was calculated for the sub sample of pairings in which both values exceeded the conventional clinical threshold of 65T.

1. Supplemental Figure S2. SRS and ADOS scores of 5 AGRE twin pairs confirmed categorically discordant by research diagnostic observation using the ADOS.


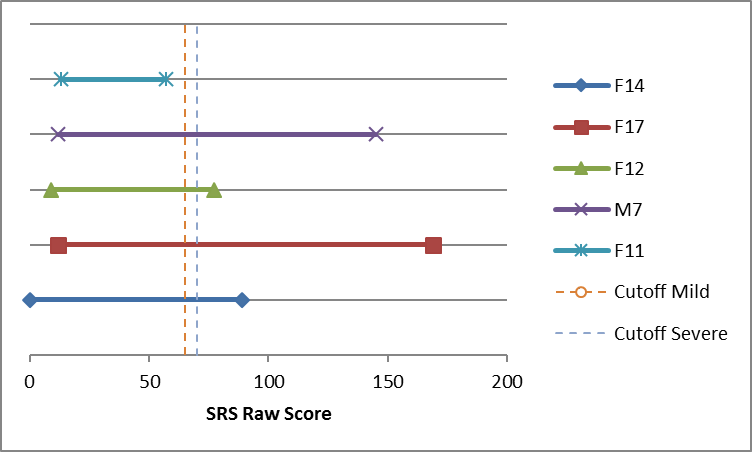

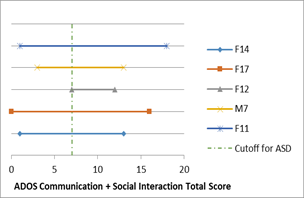


A. SRS Total

B. ADOS Communication and Social Interaction

1. Supplemental Figure S3. Scatter plots of MZ twin-co-twin data. A. General Population, Social Communication Index and Restricted Interests & Repetitive Behaviors Index; B. Clinically-ascertained, Social Communication Index and Restricted Interests & Repetitive Behaviors Index


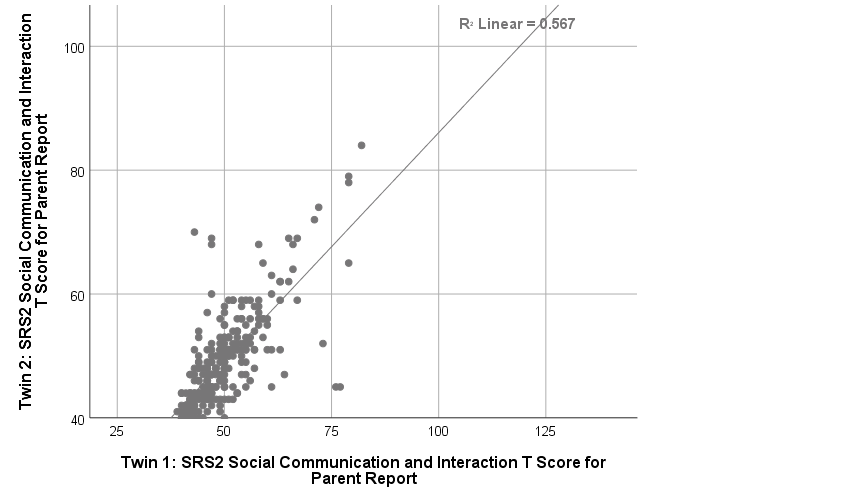

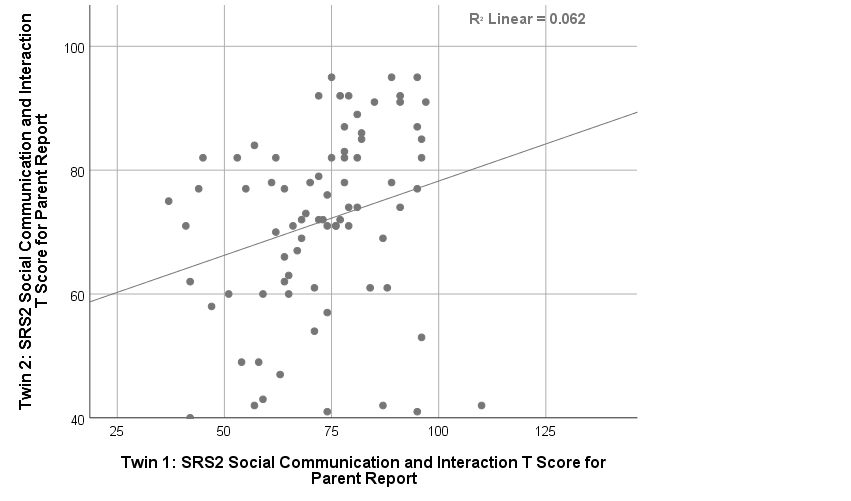


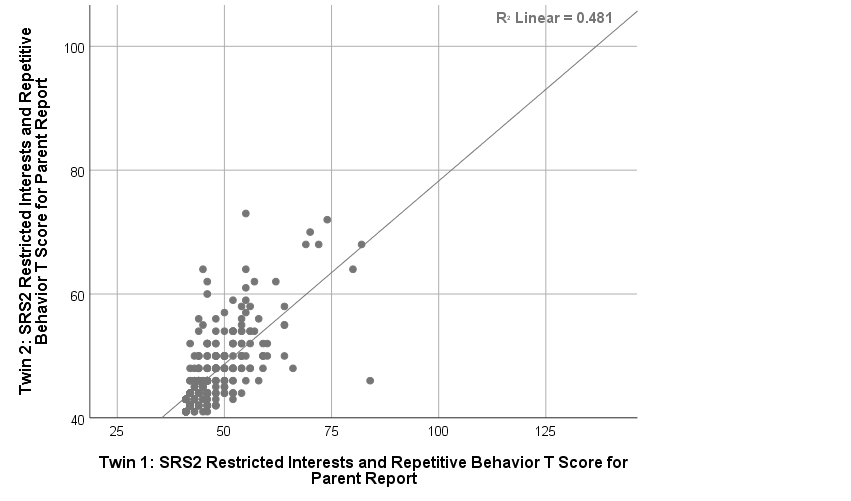

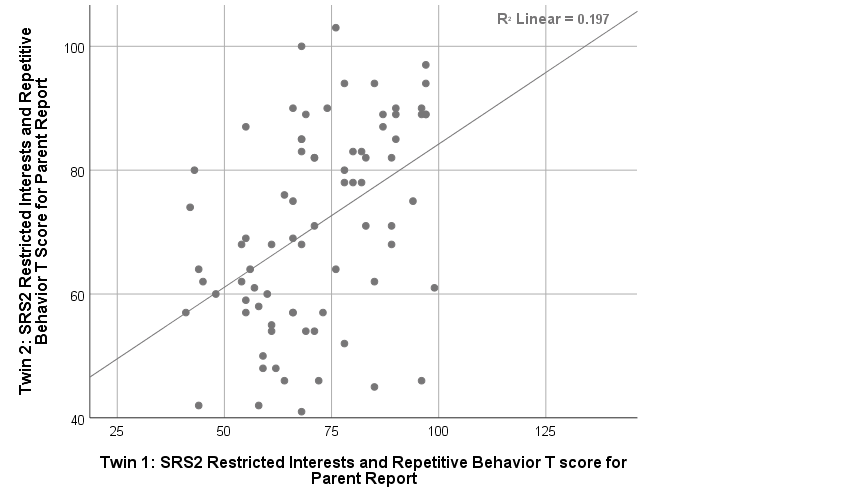


1. Supplemental Figure S4. Scatter plot of the SRS score of the higher-scoring member of each MZ twin pair vs. the SRS score difference between the MZ twins in each pair.


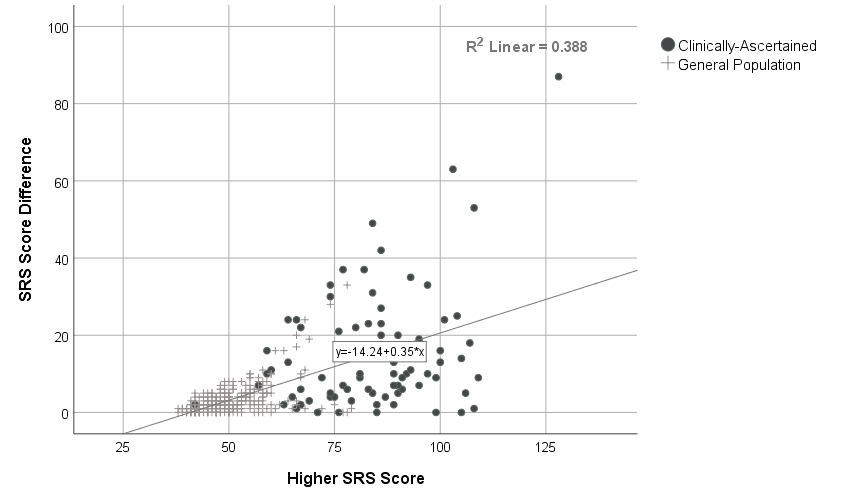


1. An illustrative case of presumed MZ twin “discordance” by community diagnosis in early childhood, clinically determined to be concordant affected MZ twins.

Three year old identical twin boys, zygosity established by molecular genetic confirmation at Washington University. One twin had received a community diagnosis for autism, while his brother was deemed “unaffected.” According to standardized ratings by parent-report, the twins fell on either side of the diagnostic threshold for clinical diagnosis. Upon comprehensive diagnostic assessment, however, it was established that the twins were concordantly affected for ASD, but with substantial differences in clinical severity. Results of a comprehensive phenotypic characterization of the twins are summarized in Table S2. We note that these twins fell just below the age range of the reported sample, but illustrate the early occurrence of marked, clinically-significant phenotypic contrasts; in this pair, restricted interests and repetitive behavior (RRB) and communicative abnormality represented the most pronounced discrepancies

Supplementary Table S2. Selected psychometric characteristics of the twins.

| **Scale** | **Subscale** | **Twin A** | **Twin B** | **Rating** |
| --- | --- | --- | --- | --- |
| **Autism Diagnostic Interview-Revised** | Total A – Qualitative Abnormalities in Reciprocal Social Interaction | 15 | 12 | Cutoff:  10 |
|  | Verbal Total B – Qualitative Abnormalities in Communication |  | 17 | Cutoff:  8 |
|  | Nonverbal Total B - Qualitative Abnormalities in Communication | 11 |  | Cutoff:  7 |
|  | Total C – Restricted, Repetitive, and Stereotyped Patterns of Behavior | 4 | 5 | Cutoff:  3 |
|  | Total D – Abnormality of Development Evident at or before 36 Months | 4 | 3 | Cutoff:  1 |
| **Autism Diagnostic Observation Schedule-2** | Overall Total | 17 | 13 |  |
|  | Social Affect Total | 13 | 8 | Cutoff: 7 |
|  | Restricted and Repetitive Behavior Total | 4 | 5 |  |
|  | Level of Autism Related Symptoms | 7 | 6 | Moderate: 5-7 |
| **Social Responsiveness Scale-2** | Total T-Score | 74 | 48 | Clinical Cutoff: 65 |
|  | Restricted Interests and Repetitive Behavior | 74 | 44 |  |
|  | Social Communication and Interaction | 49 | 41 |  |
| [**MacArthur-Bates Communicative Development Inventories**](https://mb-cdi.stanford.edu/) | Number of Words Produced | 6 | 47 | Percentile: .1 |
|  | Does the Child Use Past Tense | No | No | Percent of “Yes” answers at this child’s age: 94% |
|  | Does the Child Use Future Tense | No | Yes | Percent of “Yes” answers at this child’s age: 94% |
|  | Does the Child Use Plural | No | No | Percent of “Yes” answers at this child’s age: 93% |
|  | Does the Child Use Past Tense | No | No | Percent of “Yes” answers at this child’s age: 74% |
|  | Is the Child Combining Words | No | No | Percent of “Yes” answers at this child’s age: 99% |
| **Eye Tracking:** Proportion of time fixated on social versus non-social aspects of dynamic social scenes (Constantino et al. 2017) | Eyes | 0.40 | 0.33 |  |
|  | Mouth | 0.20 | 0.37 |  |
|  | Body | 0.25 | 0.15 |  |
|  | Object | 0.17 | 0.15 |  |

Reference

Constantino JN, Kennon-McGill S, Weichselbaum C, Marrus N, Haider A, Glowinski AL, Gillespie S, Klaiman C, Klin A, Jones W (2017) [Infant viewing of social scenes is under genetic control and is atypical in autism.](https://www.ncbi.nlm.nih.gov/pubmed/28700580) Nature 547(7663):340-344

Ramtekkar UP, Reiersen AM, Todorov AA, Todd RD (2010) Sex and age differences in attention-deficit/hyperactivity disorder symptoms and diagnoses: implications for DSM-V and ICD-11. J Am Acad Child Adolesc Psychiatry 49:217-228
